# Supplementary material for: Construction of a bioluminescence-based assay for bitter taste receptors (TAS2Rs)
Source: Sci Rep. 2022 Oct 21;12:17658. doi: 10.1038/s41598-022-21678-3 (PMC9587021; doi:10.1038/s41598-022-21678-3)
Supplement: Supplementary file 1 — Supplementary Information. [file 41598_2022_21678_MOESM1_ESM.pdf]

Supplementary Information for

Construction of a bioluminescence-based assay for bitter taste receptors (TAS2Rs)

Shi Min Tan, Wei-Guang Seetoh*

Taste Receptors Platform, Singapore Institute of Food and Biotechnology Innovation (SIFBI), Agency for Science, Technology and Research (A*STAR), 61 Biopolis Drive, Proteos, Singapore 138673

Corresponding author

*Email: seetoh_wei_guang@sifbi.a-star.edu.sg

Supplementary Table S1. Amino acid sequences of the SST_3-TAS2Rs used in this study.

SST_3 signal sequence (underlined)
Linker (in italics)
Flag tag (in bold)

Bitter taste receptor	Amino acid sequence
TAS2R3	MAAVTYPSSVPTTLDPGNASSAWPLDTSLGNASAGTSLAGLAVSG*LE*MGLTEGVFLILSGTQFTLGILVNCFIELVNGSSWFKTKRMSLSDFIITTLALLRIILLCIILTDSFLIEFSPNTHDSGIIMQIIDVSWTFTNHLSIWLATCLGVLYCLKIASFSHPTFLWLKWRVSRVMVWMLLGALLLSCGSTASLINEFKLYSVFRGIEATRNVTEHFRKKRSEYYLIHVLGTLWYLPPLIVSLASYSLLIFSLGRHTRQMLQNGTSSRDPTTEAHKRAIRIILSFFFLFLLYFLAFLIASFGNFLPKTKMAKMIGEVMTMFYPAGHSFILILGNSKLKQTFVVMLRCESGHLKPGSKGPIFS**DYKDDDDK**
TAS2R4	MAAVTYPSSVPTTLDPGNASSAWPLDTSLGNASAGTSLAGLAVSG*LE*LRLFYFSAIIASVILNFVGIIMNLFITVVNCKTWVKSHRISSSDRILFSLGITRFLMLGLFLVNTIYFVSSNTERSVYLSAFFVLCFMFLDSSSVWFVTLLNILYCVKITNFQHSVFLLLKRNISPKIPRLLLACVLISAFTTCLYITLSQASPFPELVTTRNNTSFNISEGILSLVVSLVLSSSLQFIINVTSASLLIHSLRRHIQKMQKNATGFWNPQTEAHVGAMKLMVYFLILYIPYSVATLVQYLPFYAGMDMGTKSICLIFATLYSPGHSVLIIITHPKLKTTAKKILCFKK**DYKDDDDK**
TAS2R5	MAAVTYPSSVPTTLDPGNASSAWPLDTSLGNASAGTSLAGLAVSG*LE*LSAGLGLLMLVAVVEFLIGLIGNGSLVVWSFREWIRKFNWSSYNLIILGLAGCRFLLQWLIILDLSLFPLFQSSRWLRYLSIFWVLVSQASLWFATFLSVFYCKKITTFDRPAYLWLKQRAYNLSLWCLLGYFIINLLLTVQIGLTFYHPPQGNSSIRYPFESWQYLYAFQLNSGSYLPLVVFLVSSGMLIVSLYTHHKKMKVHSAGRRDVRAKAHITALKSLGCFLLLHLVYIMASPFSITSKTYPPDLTSVFIWETLMAAYPSLHSLILIMGIPRVKQTCQKILWKTVCARRCWGP**DYKDDDDK**
TAS2R7	MAAVTYPSSVPTTLDPGNASSAWPLDTSLGNASAGTSLAGLAVSG*LE*ADKVQTTLLFLAVGEFSVGILGNAFIGLVNCMDWVKKRKIASIDLILTSLAISRICLLCVILLDCFILVLYPDVYATGKEMRIIDFFWTLTNHLSIWFATCLSIYYFFKIGNFFHPLFLWMKWRIDRVISWILLGCVVLSVFISLPATENLNADFRFCVKAKRKTNLTWSCRVNKTQHASTKLFLNLATLLPFCVCLMSFFLLILSLRRHIRRMQLSATGCRDPSTEAHVRALKAVISFLLLFIAYYLSFLIATSSYFMPETELAVIFGESIALIYPSSHSFILILGNNKLRHASLKVIWKVMSILKGRKFQQHKQI**DYKDDDDK**

TAS2R8	MAAVTYPSSVPTTLDPGNASSAWPLDTSLGNASAGTSLAGLAVSG*LE*FSPADNIFIILITGEFILGILGNGYIAL<br>VNWIDWIKKKKISTVDYILTNLVIARICLISVMVVNGIVIVLNPDVYTKNKQQIVIFTFWTFANYLNMWITTC<br>LNVFYFLKIASSSHPLFLWLKWKIDMVVHWILLGCFAISLLVSLIAAIVLSCDYRFHAIAKHKRNITEMFHVS<br>KIPYFEPLTLFNLFAIVPFIVSLISFFLLVRSLWRHTKQIKLYATGSRDPSTEVHVRAIKTMTSFIFFFFLYYISSI<br>LMTFSYLMTKYKLAVEFGEIAAILYPLGHSLILIVLNNKLRQTFVRMLTCRKIACMI**DYKDDDDK**
TAS2R9 (A187)	MAAVTYPSSVPTTLDPGNASSAWPLDTSLGNASAGTSLAGLAVSG*LE*PSAIEAIYIILIAGELTIGIWGNGFIV<br>LVNCIDWLKRRDISLIDIILISLAISRICLLCVISLDGFFMLLFPGTYGNSVLVSIVNVVWTFANNSSLWFTSCLS<br>IFYLLKIANISHPFFFWLKLKINKVMLAILLGSFLISLIISVPKNDDMWYHLFKVSHEENITWKFKVSKIPGTFK<br>QLTLNLGAMVPFILCLISFFLLLFSLVRHTKQIRLHATGFRDPSTEAHMRAIKAVIIFLLLLIVYYPVFLVMTSS<br>ALIPQGKLVLMIGDIVTVIFPSSHSFILIMGNSKLREAFLKMLRFVKCFLRRRKPFVP**DYKDDDDK**
TAS2R10	MAAVTYPSSVPTTLDPGNASSAWPLDTSLGNASAGTSLAGLAVSG*LE*LRVVEGIFIFVVVSESVFGVLGNGFI<br>GLVNCIDCAKNKLSTIGFILTGLAISRIFLIWIIITDGFIQIFSPNIYASGNLIEYISYFWVIGNQSSMWFATSLSIF<br>YFLKIANFSNYIFLWLKSRTNMVLPFMIVFLLISSLLNFAYIAKILNDYKTKNDTVWDLNMYKSEYFIKQILL<br>NLGVIFFFTLSLITCIFLIISLWRHNRQMQSNVTGLRDSNTEAHVKAMKVLISFIILFILYFIGMAIEISCFTVRE<br>NKLLLMFGMTTTAIYPWGHSFILILGNSKLKQASLRVLQQLKCCEKRKNLRVT**DYKDDDDK**
TAS2R13	MAAVTYPSSVPTTLDPGNASSAWPLDTSLGNASAGTSLAGLAVSG*LE*ESALPSIFTLVIIAEFIIGNLSNGFIVL<br>INCIDWVSKRELSSVDKLLIILAISRIGLIWEILVSWFLALHYLAIFVSGTGLRIMIFSWIVSNHFNLWLATIFSIF<br>YLLKIASFSSPAFLYLKWRVNKVILMILLGTLVFLFLNLIQINMHIKDWLDRYERNTTWNFSMSDFETFSVSV<br>KFTMTMFSLTPFTVAFISFLLLIFSLQKHLQKMQLNYKGHRDPRTKVHTNALKIVISFLLFYASFFLCVLISWI<br>SELYQNTVIYMLCETIGVFSPSSHSFLLILGNAKLRQAFLLVAAKVWAKR**DYKDDDDK**
TAS2R14	MAAVTYPSSVPTTLDPGNASSAWPLDTSLGNASAGTSLAGLAVSG*LE*GGVIKSIFTFVLIVEFIIGNLGNSFIA<br>LVNCIDWVKGRKISSVDRILTALAISRISLVWLIFGSWCVSVFFPALFATEKMFRMLTNIWTVINHFSVWLAT<br>GLGTFYFLKIANFSNSIFLYLKWRVKKVVLVLLLVTSVFLFLNIALINIHINASINGYRRNKTCSSDSSNFTRFS<br>SLIVLTSTVFIFIPFTLSLAMFLLLIFSMWKHRKKMQHTVKISGDASTKAHRGVKSVITFFLLYAIFSLSFFISV<br>WTSERLEENLIILSQVMGMAYPSCHSCVLILGNKKLRQASLSVLLWLRYMFKDGEPSGHKEFRESS**DYKDD<br>DDK**
TAS2R16	MAAVTYPSSVPTTLDPGNASSAWPLDTSLGNASAGTSLAGLAVSG*LE*IPIQLTVFFMIIYVLESLTIIVQSSLIV<br>AVLGREWLQVRRLMPVDMILISLGISRFCLQWASMLNNFCSYFNLNYVLCNLTITWEFFNILTFWLNSLLTV<br>FYCIKVSSFTHHIFLWLRWRILRLFPWILLGSLMITCVTIIPSAIGNYIQIQLLTMEHLPRNSTVTDKLENFHQY

	QFQAHTVALVIPFILFLASTIFLMASLTKQIQHHSTGHCNPSMKARFTALRSLAVLFIVFTSYFLTILITIIGTLF<br>DKRCWLWVWEAFVYAFILMHSTSLMLSSPTLKRILKGKC**DYKDDDDK**
TAS2R20	MAAVTYPSSVPTTLDPGNASSAWPLDTSLGNASAGTSLAGLAVSG*LE*MSFLHIVFSILVVVAFILGNFANGFI<br>ALINFIAWVKRQKISSADQIIAALAVSRVGLLWVILLHWYSTVLNPTSSNLKVIIFISNAWAVTNHFSIWLATS<br>LSIFYLLKIVNFSRLIFHHLKRKAKSVVLVIVLGSLFFLVCHLVMKHTYINVWTEECEGNVTWKIKLRNAMH<br>LSNLTVAMLANLIPFTLTLISFLLLIYSLCKHLKKMQLHGKGSQDPSTKIHIKALQTVTSFLILLAIYFLCLIISF<br>WNFKMRPKEIVLMLCQAFGIIYPSFHSFILIWGNKTLKQTFLSVLWQVTCWAKGQNQSTP**DYKDDDDK**
TAS2R30	MAAVTYPSSVPTTLDPGNASSAWPLDTSLGNASAGTSLAGLAVSG*LE*ITFLPIIFSILIVVIFVIGNFANGFIAL<br>VNSIEWVKRQKISFVDQILTALAVSRVGLLWVLLLHWYATQLNPAFYSVEVRITAYNVWAVTNHFSSWLA<br>TSLSMFYLLRIANFSNLIFLRIKRRVKSVVLVILLGPLLFLVCHLFVINMDETVWTKEYEGNVTWKIKLRSAM<br>YHSNMTLTMLANFVPLTLTLISFLLLICSLCKHLKKMQLHGKGSQDPSTKVHIKALQTVTSFLLLCAIYFLSM<br>IISVCNFGRLEKQPVFMFCQAIIFSYPSTHPFILILGNKKLKQIFLSVLRHVRYWVKDRSLRLHRFTRGALCVF<br>**DYKDDDDK**
TAS2R31 (WMVI)	MAAVTYPSSVPTTLDPGNASSAWPLDTSLGNASAGTSLAGLAVSG*LE*TTFIPIIFSSVVVVLFVIGNFANGFIA<br>LVNSIEWVKRQKISFADQILTALAVSRVGLLWVLLLNWYSTVFNPAFYSVEVRTTAYNVWAVTGHFSNWL<br>ATSLSIFYLLKIANFSNLIFLHLKRRVKSVILVMLLGPLLFLACQLFVINMKEIVRTKEYEGNMTWKIKLRSA<br>VYLSDATVTTLGNLVPFTLTLLCFLLLICSLCKHLKKMQLHGKGSQDPSTKVHIKVLQTVIFFLLLCAIYFLSI<br>MISVWSFGSLENKPVFMFCKAIRFSYPSIHPFILIWGNKKLKQTFLSVLRQVRYWVKGEKPSSP**DYKDDDDK**
TAS2R38 (PAV)	MAAVTYPSSVPTTLDPGNASSAWPLDTSLGNASAGTSLAGLAVSG*LE*LTLTRIRTVSYEVRSTFLFISVLEFA<br>VGFLTNAFVFLVNFWDVVKRQPLSNSDCVLLCLSISRLFLHGLLFLSAIQLTHFQKLSEPLNHSYQAIIMLWM<br>IANQANLWLAACLSLLYCSKLIRFSHTFLICLASWVSRKISQMLLGIILCSCICTVLCVWCFFSRPHFTVTTVL<br>FMNNNTRLNWQNKDLNLFYSFLFCYLWSVPPFLLFLVSSGMLTVSLGRHMRTMKVYTRNSRDPSLEAHIK<br>ALKSLVSFFCFFVISSCAAFISVPLLILWRDKIGVMVCVGIMAACPSGHAAVLISGNAKLRRAVMTILLWAQS<br>SLKVRADHKADSRTLC**DYKDDDDK**
TAS2R39	MAAVTYPSSVPTTLDPGNASSAWPLDTSLGNASAGTSLAGLAVSG*LE*LGRCFPPDTKEKQQLRMTKLCDPA<br>ESELSPFLITLILAVLLAEYLIGIIANGFIMAIHAAEWVQNKAVSTSGRILVFLSVSRIALQSLMMLEITISSTSLS<br>FYSEDAVYYAFKISFIFLNFCSLWFAAWLSFFYFVKIANFSYPLFLKLRWRITGLIPWLLWLSVFISFSHSMFCI<br>NICTVYCNNSFPIHSSNSTKKTYLSEINVVGLAFFFNLGIVTPLIMFILTATLLILSLKRHTLHMGSNATGSNDP<br>SMEAHMGAIKAISYFLILYIFNAVALFIYLSNMFDINSLWNNLCQIIMAAYPASHSILLIQDNPGLRRAWKRL

	QLRLHLYPKEWTL**DYKDDDDK**
TAS2R43	MAAVTYPSSVPTTLDPGNASSAWPLDTSLGNASAGTSLAGLAVSG*LE*ITFLPIIFSSLVVVTFVIGNFANGFIALVNSIEWFKRQKISFADQILTALAVSRVGLLWVLLLNWYSTVLNPAFNSVEVRTTAYNIWAVINHFSNWLATTLSIFYLLKIANFSNFIFLHLKRRVKSVILVMLLGPLLFLACHLFVINMNEIVRTKEFEGNMTWKIKLKSAMYFSNMTVTMVANLVPFTLTLLSFMLLICSLCKHLKKMQLHGKGSQDPSTKVHIKALQTVISFLLLCAIYFLSIMISVWSFGSLENKPVFMFCKAIRFSYPSIHPFILIWGNKKLKQTFLSVFWQMRYWVKGEKTSSP**DYKDDDDK**
TAS2R46	MAAVTYPSSVPTTLDPGNASSAWPLDTSLGNASAGTSLAGLAVSG*LE*ITFLPIIFSILIVVTFVIGNFANGFIALVNSIEWFKRQKISFADQILTALAVSRVGLLWVLVLNWYATELNPAFNSIEVRITAYNVWAVINHFSNWLATSLSIFYLLKIANFSNLIFLHLKRRVKSVVLVILLGPLLFLVCHLFVINMNQIIWTKEYEGNMTWKIKLRSAMYLSNTTVTILANLVPFTLTLISFLLLICSLCKHLKKMQLHGKGSQDPSMKVHIKALQTVTSFLLLCAIYFLSIIMSVWSFESLENKPVFMFCEAIAFSYPSTHPFILIWGNKKLKQTFLSVLWHVRYWVKGEKPSSS**DYKDDDDK**
TAS2R50	MAAVTYPSSVPTTLDPGNASSAWPLDTSLGNASAGTSLAGLAVSG*LE*ITFLYIFFSILIMVLFVLGNFANGFIALVNFIDWVKRKKISSADQILTALAVSRIGLLWALLLNWYLTVLNPAFYSVELRITSYNAWVVTNHFSMWLAANLSIFYLLKIANFSNLLFLHLKRRVRSVILVILLGTLIFLVCHLLVANMDESMWAEEYEGNMTGKMKLRNTVHLSYLTVTTLWSFIPFTLSLISFLMLICSLCKHLKKMQLHGEGSQDLSTKVHIKALQTLISFLLLCAIFFLFLIVSVWSPRRLRNDPVVMVSKAVGNIYLAFDSFILIWRTKKLKHTFLLILCQIRC**DYKDDDDK**

Supplementary Table S2. Putative signal sequences of 55 non-olfactory Class A GPCRs used in the study.

Gene name	Protein name	Receptor	Putative signal sequence
ADRA2A	Alpha-2A adrenergic receptor	α_{2A}	MGSLQPDAGNASWNGTEAPGGGARATPYSLQVT
BDKRB2	B2 bradykinin receptor	B_2	MLNITSQVLAPALNGSVSQSSGCPNTEWSGWLNVIQ
C5AR1	C5a anaphylatoxin chemotactic receptor 1	$C5a_1$	MDSFNYTTPDYGHYDDKDTLDLNTPVDKTSNT
CCKBR	Gastrin/cholecystokinin type B receptor	CCK_2	MDLLKLNRSLQGPGPGSGSSLCRPGVSLLNSSSAGNLSCETPRIRGTGTRELELTIR
CCR1	C-C chemokine receptor type 1	CCR1	METPNTTEDYDTTTEFDYGDATPCQKVNERAFGA
CCR10	C-C chemokine receptor type 10	CCR10	MGTEATEQVSWGHYSGDEEDAYSAEPLPELCYKADVQAFSRAFQPSVSLTVA
CCR2	C-C chemokine receptor type 2	CCR2	MLSTSRSRFIRNTNESGEEVTTFFDYDYGAPCH
CCR3	C-C chemokine receptor type 3	CCR3	MTTSLDTVETFGTTSYYDDVGLLCEKADTRALMA
CCR4	C-C chemokine receptor type 4	CCR4	MNPTDIADTTLDESIYSNYYLYESIPKPCTKEGIKAF
CCR6	C-C chemokine receptor type 6	CCR6	MSGESMNFSDVFDSSEDYFVSVNTSYYSVDSEMLLCSLQEVRQFSRL
CCR8	C-C chemokine receptor type 8	CCR8	MDYTLDLSVTTVTDYYYPDIFSSPCDAELIQTNGK
CHRM3	Muscarinic acetylcholine receptor M3	M_3	MTLHSNSTTSPLFPNISSSWVHSPSEAGLPLGTVTQLGSYNISQETGNFSSNDTSSDPLG
CX3CR1	CX3C chemokine receptor 1	CX_3CR1	MSTSFPELDLENFEYDDSAEACYLGDIVAFGT
CXCR1	C-X-C chemokine receptor type 1	CXCR1	MSNITDPQMWDFDDLNFTGMPPADEDYSPCMLETETLNK
CXCR2	C-X-C chemokine receptor type 2	CXCR2	MEDFNMESDSFEDFWKGEDLSNYSYSSTLPPFLLDAAPCEPESLEINK
CXCR3	C-X-C chemokine receptor type 3	CXCR3	MVLEVSDHQVLNDAEVAALLENFSSSYDYGENESDSCCTSPPCPQDFSLNFDR
CXCR5	C-X-C chemokine receptor type 5	CXCR5	MNYPLTLEMDLENLEDLFWELDRLDNYNDTSLVENHLCPATEGPLMASFKAVFV
CXCR6	C-X-C chemokine receptor type 6	CXCR6	MAEHDYHEDYGFSSFNDSSQEEHQDFLQFSKV
GALR1	Galanin receptor type 1	GAL_1	MELAVGNLSEGNASWPEPPAPEPGPLFGIGVENFVT
GNRHR	Gonadotropin-releasing hormone receptor	$GnRH_1$	MANNASLEQDPNHCSAINNSIPLIQGKLPTLTVSGKIR
GPER1	G-protein coupled estrogen receptor 1	GPER	MDVTSQARGVGLEMYPGTAQPAAPNTTSPELNLSHPLLGTALANGTGELSEHQQYVIG LFLS
GPR12	G-protein coupled receptor 12	GPR12	MNEDLKVNLSGLPRDYLDAAAAENISAAVSSRVPAVEPEPELVVNPW
GPR182	G-protein coupled receptor 182	GPR182	MSVIPSSRPVSTLAPDNDFREIHNWTELLHLFNQTFSDCHMELNENTKQVVLF
GPR20	G-protein coupled receptor 20	GPR20	MPSALSMRPWDAALPNTTAAAWTNGSVPEMPLFHHFARLDEELQAT
GRPR	Gastrin-releasing peptide receptor	BB_2	MAPNNCSHLNLDVDPFLSCNDTFNQSLSPPKMDNWFHPG
HCRTR1	Orexin receptor type 1	OX_1	MEPSATPGPQMGVPTGVGDPSLVPPDYEEEFLSYLWRDYLYPKQYE
HRH3	Histamine H3 receptor	H_3	MERAPPDGPLNASGALAGEAAAAGGARGFSAAWTAVLAA

HTR1A	5-hydroxytryptamine receptor 1A	5-HT_{1A}	MDVLSPGQGNNTTSPPAPFETGGNTTGISDVTVSYQ
HTR1B	5-hydroxytryptamine receptor 1B	5-HT_{1B}	MEEPGAQCAPPPPAGSETWVPQANLSSAPSQNCSAKD
HTR1D	5-hydroxytryptamine receptor 1D	5-HT_{1D}	MSPLNQSAEGLPQEASNRSLNATETSEAWDPRTLQAL
HTR2A	5-hydroxytryptamine receptor 2A	5-HT_{2A}	MDILCEENTSLSSTTNSLMQLNDDTRLYSNDFNSGEANTSDAFNWTVDSENRTNLSCE GCLSPSCLSLL
HTR2B	5-hydroxytryptamine receptor 2B	5-HT_{2B}	MALSYRVSELQSTIPEHILQSTFVHVISSNWSGLQTESIPEEMKQIV
KISS1R	KiSS-1 receptor	kisspeptin	MAAEATLGPNVSWWAPSNASGCPGCGVNASDGPGSAPRPLDAWLVP
MAS1L	Mas-related G-protein coupled receptor	MAS1L	MVWGKICWFSQRAGWTVFAESQISLSCSLCLHSGDQEAQNPNLVSQLCGVFLQNETN
MC3R	Melanocortin receptor 3	MC_3	MNSSCCLSSVSPMLPNLSEHPAAPPASNRSGSGFCEQ
MLNR	Motilin receptor	motilin	MGSPWNGSDGPEGAREPPWPALPPCDERRCSPFPL
MRGPRD	Mas-related G-protein coupled receptor member D	MRGPRD	MNQTLNSSGTVESALNYSRGSTVHTAYLVLSSL
MRGPRX2	Mas-related G-protein coupled receptor member X2	MRGPRX2	MDPTTPAWGTESTTVNGNDQALLLLCGKETLIP
MTNR1B	Melatonin receptor type 1B	MT_2	MSENGSFANCCEAGGWAVRPGWSGAGSARPSRTPRPP
NPBWR1	Neuropeptides B/W receptor type 1	$NPBW_1$	MHNLSLFEPGRGNVSCGGPFLGCPNESNPAPLPLPQPLA
NPSR1	Neuropeptide S receptor	NPS	MPANFTEGSFDSNGTGQMLDSSPVACTETVTFTEVVEGKEWGSFYYSFKTEQ
NPY1R	Neuropeptide Y receptor type 1	Y_1	MNSTLFSKVENHSIHYNASENSPLLAFENDDCH
NPY2R	Neuropeptide Y receptor type 2	Y_2	MGPIGAEADENQTVEEMKVEQYGPQTTPRGELVPDPEPELIDSTKLIEVQV
OPN5	Opsin-5	OPN5	MALNHTALPQDERLPHYLRDGDPFASKLSWEAD
OPRL1	Nociceptin receptor	NOP	MEPLFPAPFWEVIYGSHLQGNLSLLSPNHSLLPPHLLLNASHGAFLPL
OXGR1	2-oxoglutarate receptor 1	oxoglutarate	MIETLDSPANDSDFLDYITALENCTDEQISFKMQYLP
P2RY13	P2Y purinoceptor 13	$P2Y_{13}$	MTAAIRRQRELSILPKVTLEAMNTTVMQGFNRSERCPRDTRIVQLVFPA
PRLHR	Prolactin-releasing peptide receptor	PrRP	MASLPTQGPAAPDFFNGLLPASSSPVNQSSETVVGNGSAAGPGSQAITPFQSLQLVHQL KGL
PROKR1	Prokineticin receptor 1	PKR_1	METTMGFMDDNATNTSTSFLSVLNPHGAHATSFPFNFSYSDYDMPLDEDEDVTNSRTF FAAK
PROKR2	Prokineticin receptor 2	PKR_2	MAAQNGNASFPANFSIPQEHASSLPFNFSYDDYDLPLDEDEDMTKTQTFFAAK
PTGDR2	Prostaglandin D2 receptor 2	DP_2	MANITLKPLCPLLEEMVQLPNHSNSSLRYIDHVS
RHO	Rhodopsin	rhodopsin	MNGTEGPNFYVPFSNKTGVVRSPFEYPQYYLAE
S1PR1	Sphingosine 1-phosphate receptor 1	$S1P_1$	MGPTSVPLVKAHRSSVSDYVNYDIIVRHYNYTGKLNISADKENSIK

SSTR3	Somatostatin receptor type 3	SST_3	MAAVTYPSSVPTTLDPGNASSAWPLDTSLGNASAGTSLAGLAVSG
SSTR5	Somatostatin receptor type 5	SST_5	MEPLSLTSTPSWNASAASSSSHNWSLVDPVSPMGA

Supplementary Table S3. Amino acid sequences of the HiBiT-tagged TAS2R constructs used in the cell surface expression study.

Signal sequence (underlined, where X denotes amino acid sequence of the signal sequence in Supplementary Table S2)
Linker (in italics)
HiBiT tag (in bold)

Bitter taste receptor	Amino acid sequence
TAS2R20	X*EFGGGSGGSSSGG***VSGWRLFKKIS***GGSGGGGSGGSSSGGVD*MSFLHIVFSILVVVAFILGNFANGFIALINFIAWVKRQKISSADQIIAALAVSRVGLLWVILLHWYSTVLNPTSSNLKVIIFISNAWAVTNHFSIWLATSLSIFYLLKIVNFSRLIFHHLKRKAKSVVLVIVLGSLFFLVCHLVMKHTYINVWTEECEGNVTWKIKLRNAMHLSNLTVAMLANLIPFTLTLISFLLLIYSLCKHLKKMQLHGKGSQDPSTKIHIKALQTVTSFLILLAIYFLCLIISFWNFKMRPKEIVLMLCQAFGIIYPSFHSFILIWGNKTLKQTFLSVLWQVTCWAKGQNQSTP
TAS2R38 (PAV)	X*EFGGGSGGSSSGG***VSGWRLFKKIS***GGSGGGGSGGSSSGGVD*LTLTRIRTVSYEVRSTFLFISVLEFAVGFLTNAFVFLVNFWDVVKRQPLSNSDCVLLCLSISRLFLHGLLFLSAIQLTHFQKLSEPLNHSYQAIIMLWMIANQANLWLAACLSLLYCSKLIRFSHTFLICLASWVSRKISQMLLGIILCSCICTVLCVWCFFSRPHFTVTTVLFMNNNTRLNWQNKDLNLFYSFLFCYLWSVPPFLLFLVSSGMLTVSLGRHMRTMKVYTRNSRDPSLEAHIKALKSLVSFFCFFVISSCAAFISVPLLILWRDKIGVMVCVGIMAACPSGHAAVLISGNAKLRRAVMTILLWAQSSLKVRADHKADSRTLC
TAS2R50	X*EFGGGSGGSSSGG***VSGWRLFKKIS***GGSGGGGSGGSSSGGVD*ITFLYIFFSILIMVLFVLGNFANGFIALVNFIDWVKRKKISSADQILTALAVSRIGLLWALLLNWYLTVLNPAFYSVELRITSYNAWVVTNHFSMWLAANLSIFYLLKIANFSNLLFLHLKRRVRSVILVILLGTLIFLVCHLLVANMDESMWAEEYEGNMTGKMKLRNTVHLSYLTVTTLWSFIPFTLSLISFLMLICSLCKHLKKMQLHGEGSQDLSTKVHIKALQTLISFLLLCAIFFLFLIVSVWSPRRLRNDPVVMVSKAVGNIYLAFDSFILIWRTKKLKHTFLLILCQIRC

Supplementary Table S4. EC_{50} values of TAS2R agonists. Values indicate the mean ± s.e.m. (n = 2-4).

**Bitter Receptor**	**Compound**	**EC_{50} values**		**Published EC_{50} values**
		**293AD**	**AD-293**	
TAS2R3	Chloroquine diphosphate	45 ± 14 μM	-	172 ± 29 μM[1]
TAS2R4	Stevioside	7 ± 2 mM	7 ± 3 mM	341 ± 34 μM[2]
TAS2R5	Epigallocatechin gallate	47 ± 11 μM	-	12.3 ± 3.63 μM[3]
TAS2R7	Calcium chloride	5 ± 0.5 mM	4 ± 1 mM	5.27 ± 0.5 mM[4]
	Magnesium chloride	-	75 ± 9 mM	6.07 ± 1.07 mM[4]<br>10 ± 19.6 mM[5]
	Zinc sulphate	-	83 ± 11 mM	33.36 ± 0.14 mM[4]
	Manganese (II) chloride	-	28 ± 5 mM	6.59 ± 1.73 mM[4]<br>10 ± 1.7 mM[5]
TAS2R8	Chloramphenicol	18 ± 2.7 μM	70 ± 12 μM	41 μM[6]
	Denatonium benzoate	-	1 ± 0.1 mM	-
	Sucralose	-	11 ± 4 mM	-
TAS2R9	Pirenzepine	4 ± 0.4 mM	-	1.8 mM[7]
TAS2R10	Brucine	21 ± 6.5 μM	42 ± 3 μM	-
TAS2R13	Oxyphenonium	161 ± 16 μM	-	-
TAS2R14	Flufenamic acid	422 ± 96 nM	1,490 ± 481 nM	137 ± 17 nM[1]<br>238 ± 12.9 nM[8]
	Aristolochic acid	2.2 ± 1 μM	-	-
	Picrotoxinin	54 ± 8.4 μM	-	2.6 μM[9]<br>13.16 ± 0.93 μM[10]<br>18 μM[11]

TAS2R16	Salicin	0.4 ± 0.2 mM	2 ± 0.6 mM	0.8 ± 0.2 mM[12]<br>1.4 ± 0.2 mM[1]<br>0.417 mM[13]<br>0.22 mM[14]
	Helicin	-	3 ± 0.2 mM	2.3 ± 0.4 mM[1,15]
	Arbutin	-	5 ± 1.2 mM	5.5 ± 1.9 mM[12]<br>5.8 ± 0.9 mM[1]<br>1.34 mM[14]
	Sinigrin	-	70 ± 18 mM	0.23 mM[16]
	Phenyl β-D-glucopyranoside	-	2 ± 0.1 mM	1.1 ± 0.1 mM[15]<br>0.38 mM[14]
TAS2R20	Cromolyn	35 ± 7 μM	73 ± 26 μM	45 ± 25 μM[1]<br>64.37 ± 13 μM[17]
TAS2R30	Amarogentin	3 ± 1 μM	-	-
TAS2R31	Aristolochic acid	186 ± 70 nM	-	455 ± 5.3 nM[1]<br>130 ± 10 nM[18]<br>240 nM (WMVI)[19]<br>810 nM (RLAV)[19]
TAS2R38	Propylthiouracil	0.9 ± 0.3 μM	6 ± 2 μM	2.1 ± 0.9 μM[1]<br>1.5 μM[20]<br>2.2 μM[21]
	Phenylthiocarbamide	-	2 ± 0.4 μM	1.1 ± 0.5 μM[1]<br>6 μM[22]<br>4.5 μM[20]<br>2.3 μM[21]
	N-acetylthiourea	-	16 ± 8 μM	25 ± 16 μM[1]
	Dimethyl thioformamide	-	79 ± 12 μM	59 ± 17 μM[1]

TAS2R39	Epigallocatechin gallate	141 ± 44 µM	362 ± 81 µM	8.50 ± 2.84 µM[3]<br>161 µM[23]<br>181.6 µM[24]
TAS2R43	Aristolochic acid	20 ± 4 nM	26 ± 3 nM	8 nM[19]<br>81 ± 0.8 nM[1]
	Aloin	-	5 ± 3 µM	1.2 µM[19]<br>2.8 ± 0.4 µM[1]<br>35 µM[21]
	Caffeine	-	0.39 ± 0.12 mM	0.94 ± 0.14 mM[25]
TAS2R46	Strychnine	309 ± 73 nM	1,201 ± 433 nM	0.39 ± 0.08 µM[26]<br>0.43 ± 0.02 µM[27]<br>3.47 µM[28]
TAS2R50	Andrographolide	2 ± 0.3 µM	16 ± 3 µM	22.9 ± 4.9 µM[1]

Supplementary Table S5. Projected EC_{50} of compounds that produced a partial dose-response curve when tested in transfected cells expressing both Gα16-gust44 and mt-clytin II.

**Compound**	**EC_{50} value**
Brucine	2.3 M
Chloroquine diphosphate	> 5 M
Calcium chloride	34 mM
Zinc sulphate	2.8 M
Sinigrin	>5 M

Supplementary Table S6. Summary of the predicted length of extracellular N-terminus and *N*-glycosylation sites in TAS2Rs.

UniProt ID	Bitter taste receptor	Predicted length of extracellular N-terminus (number of amino acid residues)	*N*-glycosylation sites (amino acid position)*
Q9NYW7	TAS2R1	9	163
Q9NYW6	TAS2R3	6	166
Q9NYW5	TAS2R4	9	164, 165, 169
Q9NYW4	TAS2R5	1	155
Q9NYW3	TAS2R7	9	167, 175
Q9NYW2	TAS2R8	7	167
Q9NYW1	TAS2R9	9	164
Q9NYW0	TAS2R10	6	92, 158
Q9NYV9	TAS2R13	7	162, 166
Q9NYV8	TAS2R14	7	153, **162**, **171**
Q9NYV7	TAS2R16	1	80, **163**
P59542	TAS2R19	1	161
P59543	TAS2R20	6	161, 176
P59541	TAS2R30	1	161, 176
P59538	TAS2R31	2	161
P59533	TAS2R38	17	89, 178
P59534	TAS2R39	30	185, 194
P59535	TAS2R40	14	170, 179
P59536	TAS2R41	7	167
Q7RTR8	TAS2R42	7	163
P59537	TAS2R43	1	161, 176
P59539	TAS2R45	1	161
P59540	TAS2R46	1	**161**, 176
P59544	TAS2R50	1	161
P59551	TAS2R60	7	179

*Numbers in bold indicate Asn residues that were reported to be *N*-glycosylated.[29]

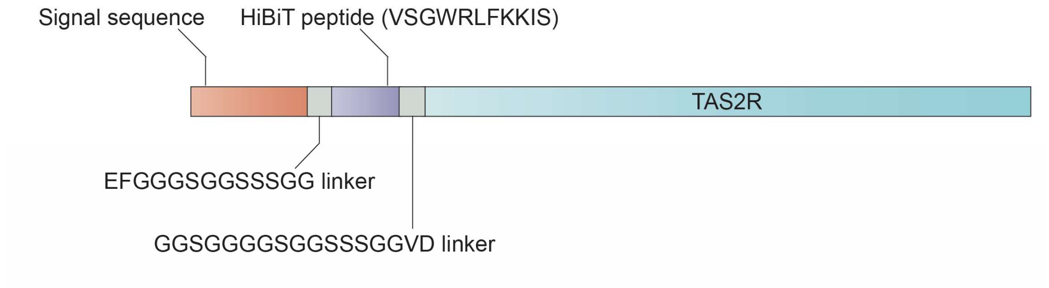

Supplementary Figure S1. The TAS2R construct used in the cell surface expression HiBiT assay consists of an N-terminal signal sequence fused to a HiBiT peptide, which is flanked by EFGGGSGGSSSGG and GGSGGGGSGGSSSGGVD linkers. This figure was generated using Adobe Illustrator (Version 25.4.1).

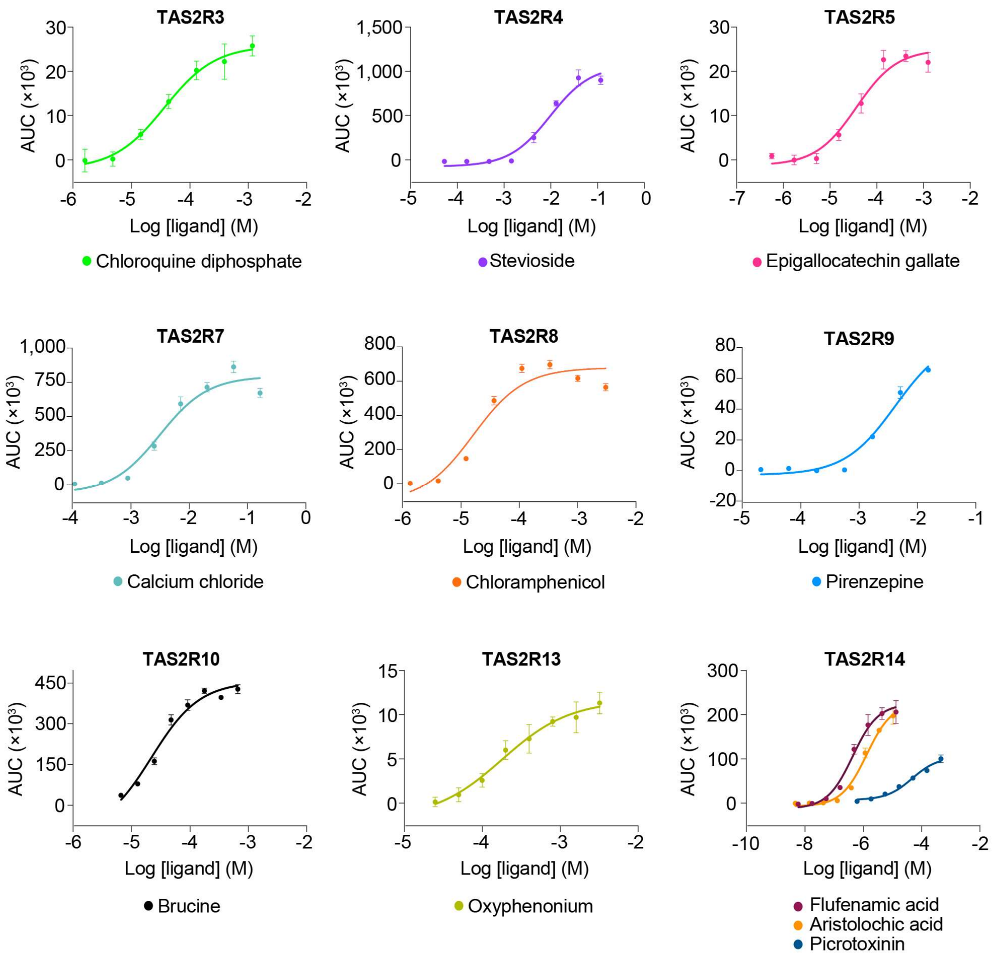

Supplementary Figure S2. (continued)

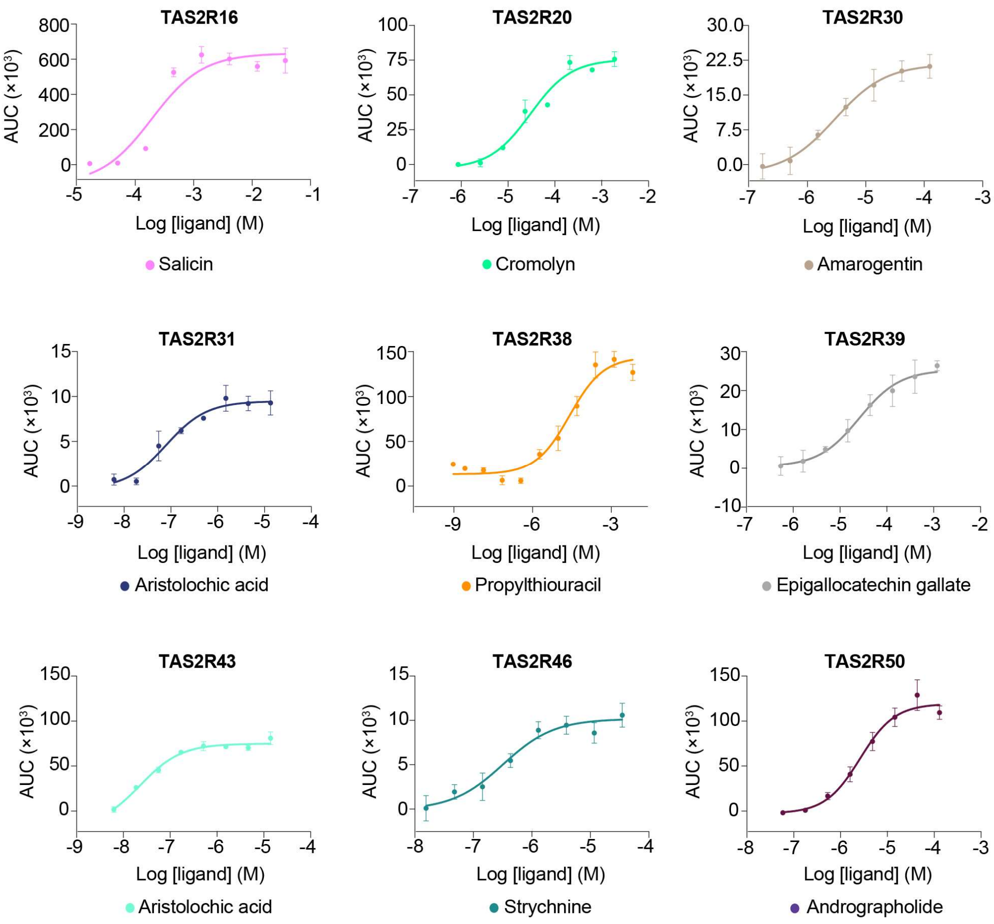

Supplementary Figure S2. Concentration-response curves of TAS2Rs upon stimulation with their cognate agonists in the bioluminescence-based intracellular calcium release assay in 293AD cells. Data points are shown as mean ± s.e.m. from a representative experiment out of three independent biological replicates performed in technical quadruplicates.

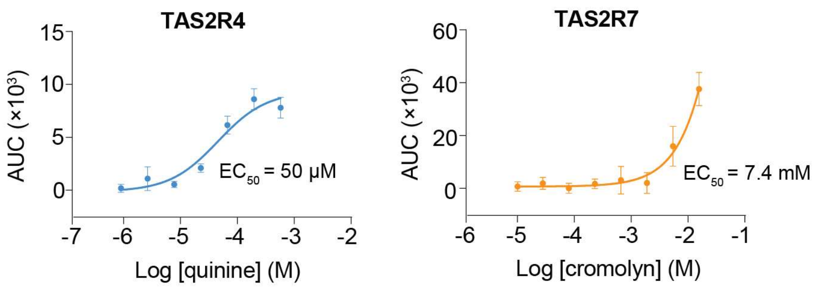

Supplementary Figure S3. Concentration-response curves of TAS2R4/7 upon stimulation with their agonists in the bioluminescence-based intracellular calcium release assay in AD-293 cells. The potency value of cromolyn (EC_{50} = 7.4 mM) obtained was similar to that reported in literature (EC_{50} = 5.9-6.67 mM).[4] While no potency value was reported for quinine against TAS2R4, our experimentally derived potency value (EC_{50} = 50 μM) was close to its reported minimal effective concentration (10 μM) that elicited response from TAS2R4-expressing cells.[1] Data points are shown as mean ± s.e.m. from a representative experiment out of two independent biological replicates performed in technical quadruplicates.

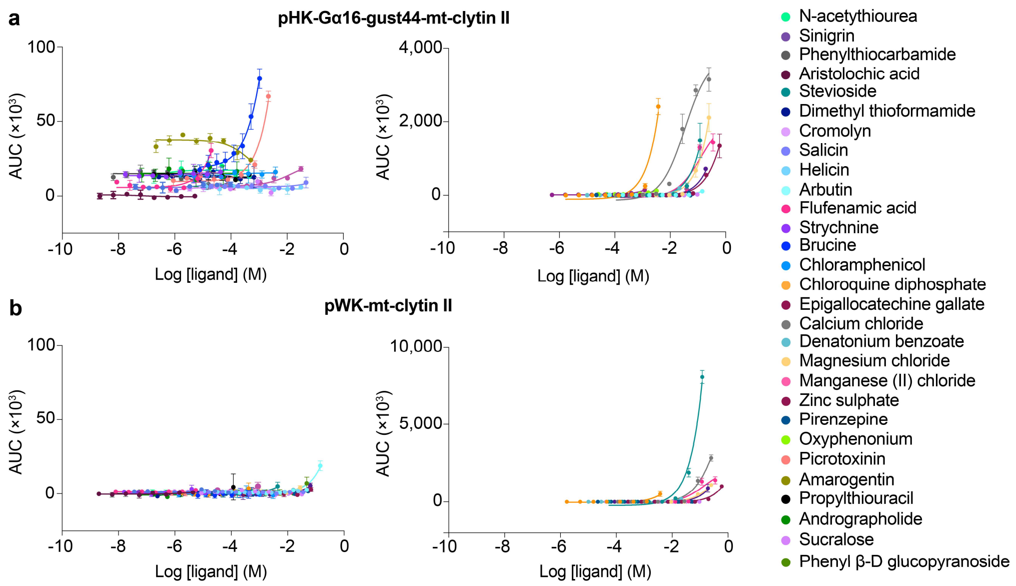

Supplementary Figure S4. Concentration-response curves of TAS2R agonists in cells transfected to express either a) Gα16-gust44 and mt-clytin II, or b) solely mt-clytin II. Data points are shown as mean ± s.e.m. from a representative experiment out of three independent biological replicates performed in technical quadruplicates.

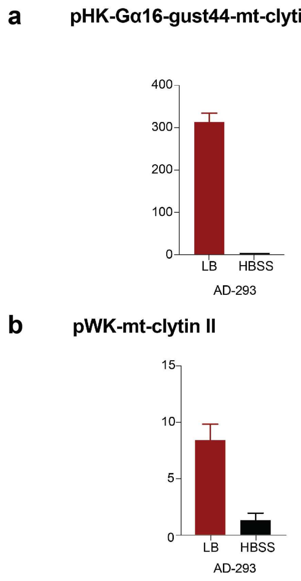

Supplementary Figure S5. Activation of calcium responses by LB medium and HBSS in AD-293 cells transfected to express a) both Gα16-gust44 and mt-clytin II, or b) solely mt-clytin II. Data points are shown as mean ± s.e.m. from a representative experiment out of two independent biological replicates performed in technical quadruplicates.

References

1. Meyerhof, W. *et al.* The molecular receptive ranges of human TAS2R bitter taste receptors. *Chem. Senses* **35**, 157–170 (2009).
2. Hellfritsch, C., Brockho, A., Sta, F., Meyerhof, W. & Hofmann, T. Human psychometric and taste receptor responses to steviol glycosides. *J. Agric. Food Chem.* **60**, 6781–6793 (2012).
3. Soares, S. *et al.* Human bitter taste receptors are activated by different classes of polyphenols. *J. Agric. Food Chem.* **66**, 8814–8823 (2018).
4. Wang, Y. *et al.* Metal ions activate the human taste receptor TAS2R7. *Chem. Senses* **44**, 339–347 (2019).
5. Behrens, M., Redel, U., Blank, K. & Meyerhof, W. The human bitter taste receptor TAS2R7 facilitates the detection of bitter salts. *Biochem. Biophys. Res. Commun.* **512**, 877–881 (2019).
6. Fotsing, J. R. *et al.* Discovery and development of S6821 and S7958 as potent TAS2R8 antagonists. *J. Med. Chem.* **63**, 4957–4977 (2020).
7. Dotson, C. D. *et al.* Bitter taste receptors influence glucose homeostasis. *PLoS One* **3**, e3974 https://doi.org/10.1371/journal.pone.0003974 (2008).
8. Di Pizio, A. *et al.* Rational design of agonists for bitter taste receptor TAS2R14: from modeling to bench and back. *Cell. Mol. Life Sci.* **77**, 531–542 (2020).
9. Yamazaki, T., Narukawa, M., Mochizuki, M., Misaka, T. & Watanabe, T. Activation of the hTAS2R14 human bitter-taste receptor by (-)-epigallocatechin gallate and (-)-epicatechin gallate. *Biosci. Biotechnol. Biochem.* **77**, 1981–1983 (2013).
10. Nowak, S. *et al.* Reengineering the ligand sensitivity of the broadly tuned human bitter taste receptor TAS2R14. *Biochim. Biophys. Acta - Gen. Subj.* **1862**, 2162–2173 (2018).
11. Behrens, M. *et al.* The human taste receptor hTAS2R14 responds to a variety of different bitter compounds. *Biochem. Biophys. Res. Commun.* **319**, 479–485 (2004).
12. Soranzo, N. *et al.* Positive selection on a high-sensitivity allele of the human bitter-taste receptor TAS2R16. *Curr. Biol.* **15**, 1257–1265 (2005).
13. Kim, M. J., Son, H. J., Kim, Y., Misaka, T. & Rhyu, M. R. Umami-bitter interactions: The suppression of bitterness by umami peptides via human bitter taste receptor. *Biochem. Biophys. Res. Commun.* **456**, 586–590 (2015).
14. Sakurai, T. *et al.* Characterization of the β-D-glucopyranoside binding site of the human bitter taste receptor hTAS2R16. *J. Biol. Chem.* **285**, 28373–28378 (2010).
15. Bufe, B., Hofmann, T., Krautwurst, D., Raguse, J. D. & Meyerhof, W. The human TAS2R16 receptor mediates bitter taste in response to β-glucopyranosides. *Nat. Genet.* **32**, 397–401 (2002).
16. Ji, M. *et al.* Identification of novel compounds for human bitter taste receptors. *Chem. Biol. Drug Des.* **84**, 63–74 (2014).
17. Jaggupilli, A. *et al.* Chemosensory bitter taste receptors (T2Rs) are activated by multiple antibiotics. *FASEB J.* **33**, 501–517 (2019).
18. Slack, J. P. *et al.* Modulation of bitter taste perception by a small molecule hTAS2R antagonist. *Curr. Biol.* **20**, 1104–1109 (2010).
19. Pronin, A. N. *et al.* Specific alleles of bitter receptor genes influence human sensitivity to the bitterness of aloin and saccharin. *Curr. Biol.* **17**, 1403–1408

(2007).
20. Behrens, M., Gunn, H. C., Ramos, P. C. M., Meyerhof, W. & Wooding, S. P. Genetic, functional, and phenotypic diversity in TAS2R38-mediated bitter taste perception. *Chem. Senses* **38**, 475–484 (2013).
21. Sandau, M. M., Goodman, J. R., Thomas, A., Rucker, J. B. & Rawson, N. E. A functional comparison of the domestic cat bitter receptors Tas2r38 and Tas2r43 with their human orthologs. *BMC Neurosci.* **16**, 1–11 (2015).
22. Greene, T. A. *et al.* Probenecid inhibits the human bitter taste receptor TAS2R16 and suppresses bitter perception of salicin. *PLoS One* **6**, e20123 https://doi.org/10.1371/journal.pone.002012 (2011).
23. Roland, W. S. U. *et al.* Bitter taste receptor activation by flavonoids and isoflavonoids: Modeled structural requirements for activation of hTAS2R14 and hTAS2R39. *J. Agric. Food Chem.* **61**, 10454–10466 (2013).
24. Narukawa, M. *et al.* Evaluation of the bitterness of green tea catechins by a cell-based assay with the human bitter taste receptor hTAS2R39. *Biochem. Biophys. Res. Commun.* **405**, 620–625 (2011).
25. Suess, B., Brockhoff, A., Meyerhof, W. & Hofmann, T. The odorant (R)-citronellal attenuates caffeine bitterness by inhibiting the bitter receptors TAS2R43 and TAS2R46. *J. Agric. Food Chem.* **66**, 2301–2311 (2018).
26. Brockhoff, A. *et al.* Receptor agonism and antagonism of dietary bitter compounds. *J. Neurosci.* **31**, 14775–14872 (2011).
27. Brockhoff, A., Behrens, M., Massarotti, A., Appending, G. & Meyerhof, W. Broad tuning of the human bitter taste receptor hTAS2R46 to various sesquiterpene lactones, clerodane and labdane diterpenoids, strychnine, and denatonium. *J. Agric. Food Chem.* **55**, 6236–6243 (2007).
28. Kuroda, Y. *et al.* Activation of human bitter taste receptors by polymethoxylated flavonoids. *Biosci. Biotechnol. Biochem.* **80**, 2014–2017 (2016).
29. Reichling, C., Meyerhof, W. & Behrens, M. Functions of human bitter taste receptors depend on N-glycosylation. *J. Neurochem.* **106**, 1138–1148 (2008).
